# Jacaranone Derivatives with Antiproliferative Activity from *Crepis pulchra* and Relevance of This Group of Plant Metabolites

**DOI:** 10.3390/plants11060782

**Published:** 2022-03-16

**Authors:** Csilla Zsuzsanna Dávid, Norbert Kúsz, Gyula Pinke, Ágnes Kulmány, István Zupkó, Judit Hohmann, Andrea Vasas

**Affiliations:** 1 Department of Pharmacognosy, Interdisciplinary Excellence Centre, University of Szeged, Eötvös u. 6, 6720 Szeged, Hungary; davidzsuzsanna88@gmail.com (C.Z.D.); kusznorbert@gmail.com (N.K.); hohmann.judit@szte.hu (J.H.); 2 Department of Water and Environmental Sciences, Faculty of Agricultural and Food Sciences, Széchenyi István University, Var Square 2, 9200 Mosonmagyaróvár, Hungary; pinke.gyula@sze.hu; 3 Department of Pharmacodynamics and Biopharmacy, University of Szeged, Eötvös u. 6, 6720 Szeged, Hungary; kulmany.agnes@gmail.com (Á.K.); zupko.istvan@szte.hu (I.Z.); 4 Interdisciplinary Centre of Natural Products, University of Szeged, Eötvös u. 6, 6720 Szeged, Hungary

**Keywords:** jacaranones, *Crepis pulchra*, Cichorioideae, Asteraceae, antiproliferative activity

## Abstract

Jacaranones are a small group of specific plant metabolites with promising biological activities. The occurrence of jacaranones is limited to only a few plant families, with Asteraceae being the most abundant source of these compounds. Therefore, jacaranones can also serve as chemotaxonomic markers. Our phytochemical investigation of *Crepis pulchra* L. (Asteraceae) resulted in three jacaranone derivatives (jacaranone, 2,3-dihydro-2-hydroxyjacaranone, 2,3-dihydro-2-methoxyjacaranone), and (6*R*,9*S*)-3-oxo-α-ionol-*β*-d-glucopyranoside, fulgidic acid, 12,15-octadecadienoic acid methyl ester, scopoletin and apigenin-7-*O*-*β*-d-glucoside. This is the first report on the isolation of jacaranones from a species belonging to the Cichorioideae subfamily of Asteraceae. Jacaranone derivatives were subjected to an in vitro antiproliferative assay against a panel of human cancer cell lines (MCF-7, MDA-MB-231, HeLa, and C33A), revealing high or moderate activities, with IC_50_ values ranging from 6.3 to 26.5 μM.

## 1. Introduction

Jacaranone derivatives, bearing an unsaturated cyclohexanone skeleton, occur rarely in the plant kingdom. Jacaranone [methyl 2-(1-hydroxy-4-oxocyclohexa-2,5-dien-1-yl)acetate] (**2**), the methyl ester derivative of quinolacetic acid (**1**), was first isolated from *Jacaranda caucana* (Bignoniaceae) by Ogura et al. in 1976 [1]. Since then, jacaranone derivatives (*n* = 35) have been isolated from other plant species as well, most of them belonging to the family Asteraceae. Almost all of the isolated compounds have been studied for their biological activities (e.g., cytotoxic, antimicrobial, anti-inflammatory, antioxidant, sedative, antiprotozoal, and antifeedant effects), and many of them showed multiple activities [2,3,4,5,6,7,8].

The genus *Crepis* (family Asteraceae) includes about 200 annual, biennial, or perennial plant species occurring widely in Eurasia, Africa, and North America [9]. In folk medicine, decoctions prepared from aerial parts of members of the genus *Crepis* are used for the treatment of various diseases, e.g., cough (Uganda) [10]; hepatitis, jaundice, and gallstones (Yemen) [11]; tumors (USA and China) [12]; and cardiovascular diseases, diabetes, cold, catarrh, and eye diseases (Turkey) [13,14,15,16]. Some species are traditionally used for their diuretic or laxative properties (Italy) [17,18] or externally for healing wounds, bruises, or inflammation (Spain and Bangladesh) [19,20].

*Crepis* species are rich sources of guaianolide- and eudesmane-type sesquiterpenes [21,22,23] and flavonoids [24,25]. In vitro pharmacological assessments revealed that *Crepis* extracts possess hepatoprotective [11], antimicrobial [21], antiviral [26], antioxidant [27], anti-inflammatory [28], and antiproliferative [26,27] activities.

Smallflower hawksbeard (*Crepis pulchra* L.) occurs in different ruderal stands. The species can be found in dry, open habitats, grasslands, pastures, abandoned fields, waste areas, alongside railroads, and roadsides [29,30]. According to the literature data, previously, guaianolide-type sesquiterpenes (8-*epi*-isoamberboin, vernoflexuoside, macrocliniside A, and diaspanoside A) were isolated from the plant by Kisiel et al. [31].

In continuation of our research aiming at the discovery of new bioactive specific metabolites from medicinal plants, the methanol extract of *Crepis pulchra* L. was investigated. The isolated special metabolites are discussed in comparison with the literature dealing with naturally occurring jacaranones.

## 2. Results

### 2.1. Isolation of Compounds from C. pulchra

The dried and ground whole plant material (1.25 kg) was extracted with methanol at room temperature. After evaporation, the extract was dissolved in 50% aqueous methanol, and solvent–solvent partition was performed with *n*-hexane, chloroform, and ethyl acetate. The chloroform phase was purified by a combination of different methods, including column chromatography (CC), vacuum liquid chromatography (VLC), thin layer chromatography (TLC), and HPLC to afford eight compounds. The structural determination was carried out by extensive spectroscopic analysis using 1D (^1^H, JMOD) and 2D NMR (^1^H-^1^H COSY, HSQC, HMBC, NOESY) spectroscopy, HRESIMS measurements, and the comparison of the spectral data with the literature values.

**Figure 1:**
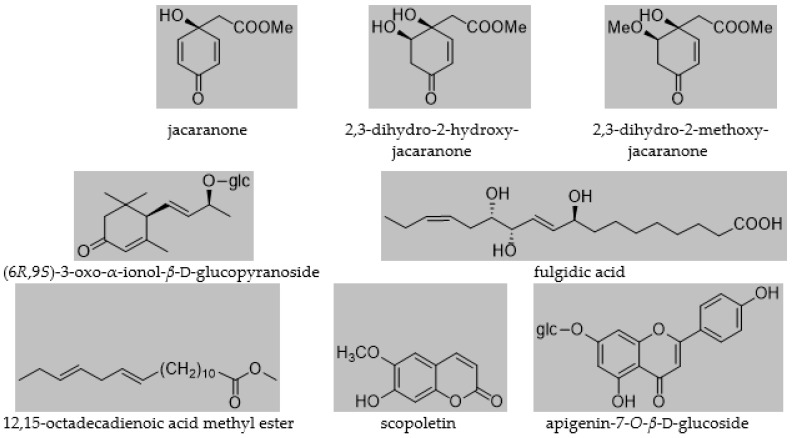
Compounds isolated from *C. pulchra*.

The isolated compounds were identified as jacaranone [5], 2,3-dihydro-2-hydroxyjacaranone [5]; 2,3-dihydro-2-methoxyjacaranone [5]; (6*R*,9*S*)-3-oxo-ionol-*β*-d-glucopyranoside [32]; fulgidic acid [33]; 12,15-octadecadienoic acid methyl ester, scopoletin [34]; and apigenin-7-*O*-*β*-d-glucoside [35] (Figure 1). All compounds were isolated for the first time from the plant. Moreover, this was the first time jacaranone derivatives were isolated from a *Crepis* species.

### 2.2. Antiproliferative Investigation of the Isolated Jacaranones

The jacaranone derivatives, isolated from *C. pulchra*, were subjected to an in vitro cytotoxicity (MTT) assay against human cancer (breast cancer (MCF-7 and MDA-MB-231), and cervical cancer (HeLa and C33A) cell lines (Table 1). Jacaranone proved to be the most active against all four tested cell lines (IC_50_ 6.27–14.61 µM). Its activity was comparable with that of the positive control, cisplatin. 2,3-Dihydro-2-hydroxyjacaranone and 2,3-dihydro-2-methoxyjacaranone differ from jacaranone only in the substitution of C-2 (a hydroxy group in the case of 2,3-dihydro-2-hydroxyjacaranone, and a methoxy group in 2,3-dihydro-2-methoxyjacaranone) and in the saturation of the double bond between C-2–C-3. These modifications resulted in the decrease of the antiproliferative activity in the case of the two jacaranone derivatives. Our results confirm that the presence of an α,β-unsaturated carbonyl group in the molecule is essential for the antiproliferative activity of jacaranones [36].

## 3. Discussion

### 3.1. Occurrence of Jacaranones in Nature

**Figure 2:**
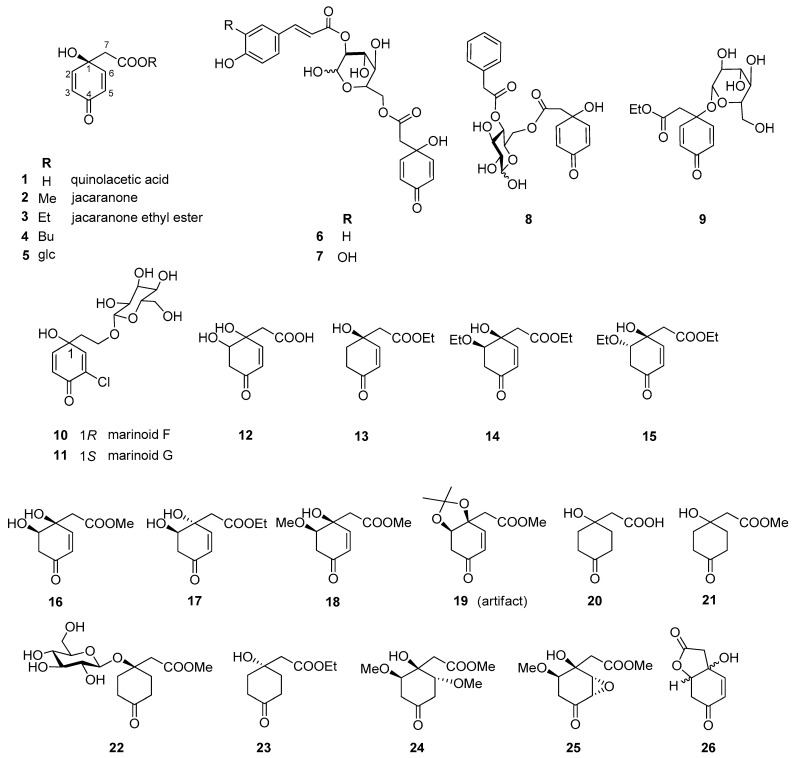
Naturally occurring jacaranone derivatives (monomers) *. * Stereochemistry of the compounds is indicated according to the source literature; Me = methyl, Et = ethyl, Bu = butyl, glc = glucose.

**Figure 3:**
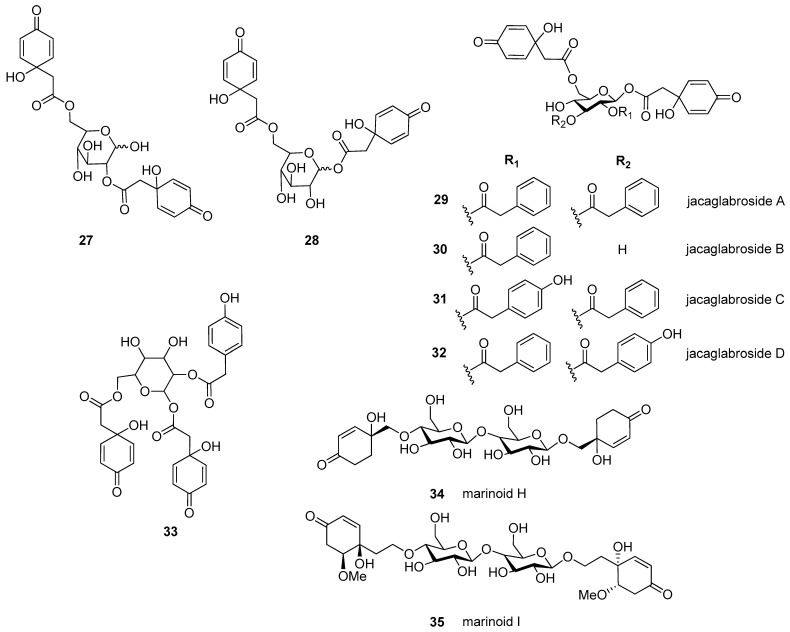
Naturally occurring jacaranone derivatives (dimers) *. * Stereochemistry of each compound is given as it was published.

Altogether, 35 jacaranones were isolated from 37 plant species; three-quarters of the compounds are monomers (*n* = 26), and the others are dimers (*n* = 9) (Figure 2 and Figure 3). Although the best sources of jacaranones are Asteraceae species, certain species of the Acanthaceae [3], Bignoniaceae [1,2,37,38,39,40,41], Delesseriaceae [42], Gesneriaceae [43,44], Oleaceae [45], and Theaceae [4,5] families were also found to be sources of jacaranones (Table 2). Among Asteraceae species, the genus *Senecio* is represented by 20 jacaranone derivative-producing plant species [36,46,47,48,49,50,51,52,53,54,55,56,57,58,59,60,61,62]. Besides the *Bethencourtia* [8], *Packera* [63], and *Pentacalia* [6,7] genera, each is represented by one species. All species belong to the Asteroideae (Tubuliflorae) subfamily of Asteraceae. *Crepis pulchra* is the first representative of the Cichorioideae (Liguliflorae) subfamily. Jacaranone (**2**) is the most common compound, isolated from 28 plant species of the Asteraceae, Bignoniaceae, and Theaceae families.

The basic monomer structure of jacaranones can be modified by different substituents. Regarding the cyclohexanone ring, substitution occurs mainly at C-2, where hydroxy- (**12**), methoxy- (**24**, **35**), or ethoxy- (**14**, **15**) groups are linked to the ring, or even an epoxide ring can be formed, as in the case of compound **25**. A rare substituent can be found in cases of marinoid F (**10**) and marinoid G (**11**), where a chlorine atom is joined to the cyclohexanone ring at C-3. Most frequently, an ester is formed via the carboxyl group with aliphatic alcohols, e.g., methanol (**2**, **16**, **18**, **21**, **22**, **24**, **25**), ethanol (**3**, **13**–**15**, **17**, **23**), or butanol (**4**), or with a sugar molecule (**5**–**8**). A sugar molecule can also be attached to the hydroxy group forming an acetal (**9**, **22**). Compound **26**, isolated from *Senecio giganteus*, is the only compound containing a lactone ring. Jacaranone dimers are formed from two monomers linked through one or two sugar molecules.

### 3.2. Antiproliferative Activity of Jacaranones

According to the relevant literature, the most promising biological effect of jacaranones is their anticancer activity. The methanolic extract of *Jacaranda caucana* and jacaranone (**2**) were tested against P-388 lymphocytic leukemia and Eagle’s 9KB carcinoma cells, and substantial antiproliferative activity was detected [1,64]. The cytotoxicity of **2** was investigated against six tumors (lung large cell carcinoma (COR-L23), colorectal adenocarcinoma (Caco-2), amelanotic melanoma (C32), hepatocellular carcinoma (HepG-2), renal cell adenocarcinoma (ACHN), and hormone dependent prostate carcinoma (LNCaP) and one normal (human fetal lung (MRC-5) cell lines [47,55]. Jacaranone (**2**) showed outstanding action against all tested cell lines (IC_50_ values ranging from 11.31 to 40.57 µM), which was comparable with that of the positive control vinblastine, while **2** did not adversely affect MRC-5 cells. Furthermore, **2** exerted antiproliferative and proapoptotic effects in eight human (A2058, SK-MEL-28, HCT-8, LS160, SiHa, HL-60, SK-BR-3) and one murine (B16F10-Nex2) tumor cell lines in vitro by downregulating Akt and activating p38 MAPK signaling pathways through the generation of reactive oxygen species (ROS). IC_50_ values varied from 9 to 145 µM for human cancer cells and was 17 µM for murine melanoma B16F10-Nex2 cells [65]. Moreover, the protective effect of this quinone (**2**) was also proved in a melanoma syngeneic model in vivo [65]. Jacaranone ethyl ester (**3**) was tested in KB screen and was found to be highly active (ED_50_ value 16.82 µM) [48]. Tian et al. investigated the cytotoxic effect of seven jacaranone analogs against various tumorous cell lines (HCT8, CaEa-17, A2780, HeLa, BEL-7402, KB, PC-3M, A549, BGC-823, and MCF-7) [59]. Jacaranone ethyl ester (**3**) possessed the most potent cytotoxic effect with IC_50_ values at a range of 2.85–4.33 µM. Fluorouracil was applied as a positive control (IC_50_s 4.15–6.84 µM). Apart from jacaranone ethyl ester (**3**), quinolacetic acid (**1**), jacaranone (**2**), and a new jacaranone-derivative (**17**) also showed relatively high cytotoxic activity. Based on the results of their cytotoxicity assay, the authors found that all the active compounds share the same structural moiety, the α,β-unsaturated carbonyl group, a segment that is known to be of crucial importance for the cytotoxic effect of other compounds as well [59].

Presser et al. synthesized 13 nitrogen-containing jacaranone derivatives from the natural-product-derived cyclohexadienone scaffold and investigated their antiproliferative activity against four human tumorous cell lines (MDA-MB-231 breast cancer, CCRF-CEM leukemia, HCT-116 colon cancer, and U251 glioblastoma), and one non-tumorigenic cell line (MRC-5 lung fibroblasts) at 5 µg/mL and 50 µg/mL concentrations [66]. The positive control vinblastine was applied at 0.01 µg/mL. At 50 µg/mL concentration, almost all derivatives were found to have cytotoxic effect against MDA-MB-231 and CCRF-CEM cells. During their investigations, the authors managed to reveal some structure–activity relationships of jacaranone-based nitrogenous cyclohexadienones as well. It was observed that the most potent compounds shared an α,β-unsaturated imide structural element. In the absence of this structural moiety, no cytotoxic effect could be detected [66].

## 4. Materials and Methods

### 4.1. General Experimental Procedures

NMR spectra were recorded in methanol *d*_4_ on a Bruker Avance DRX 500 spectrometer at 500 MHz (^1^H) and 125 MHz (^13^C). The signals of the deuterated solvents were chosen as references. The chemical shift values (δ) were given in ppm, and the coupling constants (J) are in Hz. Two-dimensional (2D) experiments were performed with standard Bruker software. In the ^1^H–^1^H COSY, HSQC, and HMBC experiments, gradient-enhanced versions were used. Column chromatography (CC) was performed on polyamide (MP Biomedicals Germany GmbH, Eschwege, Germany). Normal and reversed-phase vacuum liquid chromatography (VLC) was carried out on silica gel (Kieselgel 60 GF_254_, 15 µm, Merck, Darmstadt, Germany) and on reversed phase silica gel [RediSep C-18, 40–60 µm, Teledyne Isco, Lincoln, NE, USA]. Thin-layer chromatography was performed on a Kieselgel 60 RP-18 F_254_ and a Kieselgel 60 F_254_ (Merck, Darmstadt, Germany). Spots on UV active silica gel were detected under UV light (245 nm and 336 nm) and made visible with vanillin sulfuric acid and heating at 105 °C for 2 min. The high-performance liquid chromatographic (HPLC) separation was carried out on a Waters HPLC (Waters 600 controller, Waters 600 pump, and Waters 2998 photodiode array detector), using a normal phase (LiChrospher Si 100 (250 × 4 mm, 5 μm, Merck)) column. The flow rate was 1 mL/min, and the injection volume was 25 μL. The data were acquired and processed with Empower software. All solvents used for CC were of at least analytical grade (VWR Ltd., Szeged, Hungary). Ultrapure water was prepared with a Milli-Q water purification system (Millipore, Molsheim, France).

### 4.2. Plant Material

The whole plants of *Crepis pulchra* (1.25 kg dried plant material) were collected in the flowering period at Hegyeshalom (Hungary) in July 2019, and were identified by one of the authors, Gyula Pinke (Department of Water and Environmental Sciences, Széchenyi István University). A voucher specimen (No. 895) has been deposited at the Department of Pharmacognosy, University of Szeged, Szeged, Hungary.

### 4.3. Extraction and Isolation

The dried and ground whole plant of *C. pulchra* (1.25 kg) was percolated with methanol (12.5 L) at room temperature. The crude extract was concentrated in vacuo (152.5 g), redissolved in water, and subjected to solvent–solvent partition with *n*-hexane (5 × 500 mL), chloroform (5 × 500 mL), and ethyl acetate (5 × 400 mL), respectively. The concentrated chloroform-soluble fraction (35.7 g) was further separated by polyamide open-column chromatography with a gradient system of MeOH–H_2_O (2:3, 3:2, and 4:1 (600 mL/eluent); each eluent was collected as a fraction (Fractions I–III). Fraction I obtained with 40% MeOH (3.04 g) was subjected to vacuum liquid chromatography (VLC) on silica gel with a gradient system of CHCl_3_–MeOH (from 99:1 to 7:3 (300 mL/eluent) to yield 10 major fractions (I/1-10). Fraction I/3 (219 mg) was further purified by VLC on reversed phase silica gel using a MeOH–H_2_O gradient system (1:9 → 9:1, 100 mL/eluent) to afford 8 subfractions (I/3/1-8). Subfraction I/3/1 (159 mg) was subjected to normal phase VLC (*n*-hexane–isopropanol, gradient, 95:5 → 6:4, 25 mL/eluent), followed by reversed phase preparative TLC (using MeOH–H_2_O 1:1 as an eluent) to yield 4 (**2** (27.3 mg), **16** (4.4 mg), **18** (3.6 mg), and scopoletine (12.9 mg) compounds. Fraction I/9 (1.0 g) was separated by normal phase VLC with a gradient system of CHCl_3_–MeOH (from 98:2 to 7:3 (50 mL/eluent) to afford 10 subfractions (I/9/1-10). Fraction I/9/6 (345.5 mg) was purified by reverse-phase VLC (gradient system of MeOH–H_2_O 1:9 → 8:2, 25 mL/eluent) and gel chromatography on Sephadex LH-20 (using CH_2_Cl_2_–MeOH 1:1 as an eluent) followed by normal-phase preparative TLC with EtOAc–MeOH–H_2_O (100:16:0,5) as a solvent system to yield fulgidic acid (5.19 mg). Fraction I/9/7 (185.0 mg) was separated by gel chromatography on Sephadex LH-20 (using CH_2_Cl_2_–MeOH 1:1 as an eluent) and then by preparative TLC on reverse-phase silica gel with MeOH–H_2_O (1:1) to yield (6*R*,9*S*)-3-oxo-ionol-*β*-d-glucopyranoside (3.34 mg). Fraction II obtained with 60% MeOH (2.08 g) was subjected to reverse-phase VLC, applying a gradient system of MeOH–H_2_O (from 1:9 to 1:0, 200 mL/eluent) to obtain nine main fractions (II/1-9). Separation of fraction II/1 (236.3 mg) by gel chromatography on Sephadex LH-20 afforded five subfractions (II/1/1-5). Subfraction II/1/4 (60.8 mg) was further purified by normal-phase VLC using CHCl_3_–MeOH as a mobile phase [from pure 1:0 to 6:4 (40 mL/eluent)] followed by preparative TLC on normal-phase silica gel with EtOAc–HCOOH–H_2_O (85:10:5) as a solvent system to obtain apigenin-7-*O*-glucoside (7.8 mg). Fraction II/9 (165.1 mg) was subjected to normal-phase VLC, applying a gradient system of cyclohexane–EtOAc–MeOH [from 9:1:0 to 6:3:1 (50 mL/eluent)] to afford ten subfractions (II/9/1-10). Subfraction II/9/2 (4.4 mg) was purified by normal-phase HPLC [isocratic, cyclohexane–EtOAc (8:2)] to yield 12,15-octadecadienoic acid methyl ester (1.5 mg).

### 4.4. Antiproliferative (MTT) Assay

The growth-inhibition properties of jacaranone derivatives were determined by standard MTT assays on four human malignant gynecological cell lines (breast cancer: MCF-7 and MDA-MB-231, and cervical cancer: HeLa and C33A). All cell lines were maintained in minimal essential medium (MEM) supplemented with 10% fetal bovine serum, 1% nonessential amino acids, and 1% penicillin–streptomycin–amphotericin B mixture in humidified air containing 5% CO_2_ at 37 °C. All cell types were seeded into 96-well plates at a density of 5000 with the exception of C33A, which was seeded at a density of 10,000 and treated by increasing concentrations (0.1–30 μM) of the compounds for 72 h under cell culturing conditions. After the incubation, 5 mg/mL MTT [3-(4,5-dimethylthiazol-2-yl)-2,5-diphenyltetrazolium bromide] solution was added to samples for 4 h and precipitated blue formazan crystals were dissolved in DMSO. Absorbance values of the samples were measured at 545 nm using a microplate reader (Stat Fax-2100, Awareness Technologies Inc., Palm City, FL, USA), and untreated cells were used as a control. Normalized sigmoidal concentration−response curves were fitted to the determined data, and the IC_50_ values were calculated by GraphPad Prism 5.01 (GraphPad Software, San Diego, CA, USA). Cisplatin (Ebewe Pharma GmbH, Unterach, Austria) was used as a reference agent in the same concentration range.

## 5. Conclusions

In our experiment, a combination of different chromatographic techniques resulted in the isolation of eight compounds, among them three jacaranone derivatives from *C. pulchra* for the first time. Jacaranone (**2**) showed the highest antiproliferative activity against MDA-MB-231 (human breast cancer) and C33A (human cervical cancer) cells. Although jacaranones represent a small group of plant special metabolites, they can be interesting either for organic chemists or for pharmacologists because of their promising biological effects. Moreover, they can serve as chemotaxonomic markers. The importance of our results is that smallflower hawksbeard is the first representative of the Cichorioideae subfamily of Asteraceae in which jacaranones have been detected.

## Figures and Tables

**Table 1:** Antiproliferative activity of the isolated jacaranone derivatives.

Compound	IC_50_ (µM) [95% Confidence Intervals]			
	MCF-7	MDA-MB-231	HeLa	C33A
jacaranone	10.22 [8.96–11.66]	6.89 [6.29–7.55]	14.61 [13.40–15.93]	6.27 [5.66–6.95]
2,3-dihydro-2-hydroxyjacaranone	>30	26.49 [21.61–32.41]	>30	21.56 [18.86–24.64]
2,3-dihydro-2-methoxyjacaranone	21.30 [19.44–23.35]	17.85 [15.39–20.70]	23.47 [21.36–25.78]	12.52 [11.27–13.92]
cisplatin	6.01 [5.33–6.79]	18.65 [16.67–20.85]	14.02 [12.65–15.56]	3.69 [3.22–3.95]

**Table 2:** List of plant families and species containing jacaranones.

Family	Species	Compound	Ref.
Asteraceae	*Bethencourtia hermosa*	**3, 17**	[8]
	*Jacobaea gigantea = Senecio giganteus*	**2, 26**	[46]
	*Packera bellidifolia*	**2, 21**	[63]
	*Pentacalia desiderabilis*	**1–3, 21**	[6,7]
	*Senecio ambiguous* subsp. *ambiguus*	**2**	[47]
	*Senecio anonymus*	**3**	[48]
	*Senecio argunensis*	**2, 18, 19, 21, 24, 25**	[36]
	*Senecio cannabifolius*	**2, 3, 18, 19, 24**	[49,50]
	*Senecio cannabifolius* var. *integrilifolius*	**21**	[51]
	*Senecio carpathicus*	**2, 4**	[62]
	*Senecio clevelandii*	**2, 21**	[52]
	*Senecio chrysanoides*	**13–15, 23**	[53]
	*Senecio erucifolius*	**2, 4**	[62]
	*Senecio fendleri*	**3**	[54]
	*Senecio leucanthemifolius*	**2**	[55]
	*Senecio minutus*	**2, 24**	[56]
	*Senecio othonnae*	**2, 4**	[62]
	*Senecio palmatus*	**2**	[57]
	*Senecio paludosus*	**2, 4**	[62]
	*Senecio scandens*	**1–3, 5–7, 9, 17, 20, 27, 28**	[58,59,60]
	*Senecio scandens* var. *incisus*	**6, 7, 12**	[61]
	*Senecio subalpinus*	**2, 4**	[62]
	*Senecio wagneri*	**2, 4**	[62]
Acanthaceae	*Avicennia marina*	**10, 11, 34, 35**	[3]
Bignoniaceae	*Jacaranda arborea*	**2, 3**	[2]
	*Jacaranda caucana*	**2**	[1]
	*Jacaranda glabra*	**2, 29–32**	[37]
	*Jacaranda macrantha*	**2**	[38]
	*Jacaranda mimosifolia*	**2, 33**	[39]
	*Jacaranda oxyphylla*	**8**	[40]
	*Jacaranda puberula*	**2**	[41]
Delesseriaceae	*Delesseria sanguineu*	**2**	[42]
Gesneriaceae	*Sinningia mauroana*	**2**	[43]
	*Sinningia reitzii*	**2**	[44]
Oleaceae	*Forsythia suspensa*	**22**	[45]
Theaceae	*Ternstroemia japonica*	**2, 16, 18**	[5]
	*Ternstroemia pringlei*	**2**	[4]

## Data Availability

Not applicable.
